# Expression levels of the metalloproteinase ADAM8 critically regulate proliferation, migration and malignant signalling events in hepatoma cells

**DOI:** 10.1111/jcmm.16015

**Published:** 2020-12-13

**Authors:** Tanzeela Awan, Aaron Babendreyer, Abid Mahmood Alvi, Stefan Düsterhöft, Daniela Lambertz, Jörg W. Bartsch, Christian Liedtke, Andreas Ludwig

**Affiliations:** ^1^ Institute of Molecular Pharmacology RWTH Aachen University Aachen Germany; ^2^ Department of Medicine III University Hospital RWTH Aachen University Aachen Germany; ^3^ Department of Neurosurgery Philipps University Marburg University Hospital Marburg Marburg Germany

**Keywords:** focal adhesion kinase, hepatocellular carcinoma, integrin, metalloproteinase, migration

## Abstract

Hepatocellular carcinoma (HCC) is one of the most common metastatic tumours. Tumour growth and metastasis depend on the induction of cell proliferation and migration by various mediators. Here, we report that the A Disintegrin and Metalloproteinase (ADAM) 8 is highly expressed in murine HCC tissues as well as in murine and human hepatoma cell lines Hepa1‐6 and HepG2, respectively. To establish a dose‐dependent role of different ADAM8 expression levels for HCC progression, ADAM8 expression was either reduced *via* shRNA‐ or siRNA‐mediated knockdown or increased by using a retroviral overexpression vector. These two complementary approaches revealed that ADAM8 expression levels correlated positively with proliferation, clonogenicity, migration and matrix invasion and negatively with apoptosis of hepatoma cells. Furthermore, the analysis of pro‐migratory and proliferative signalling pathways revealed that ADAM8 expression level was positively associated with expression of β1 integrin as well as with the activation of focal adhesion kinase (FAK), mitogen‐activated protein kinase (MAPK), Src kinase and Rho A GTPase. Finally, up‐regulation of promigatory signalling and cell migration was also seen with a proteolytically inactive ADAM8 mutant. These findings reveal that ADAM8 is critically up‐regulated in hepatoma cells contributes to cell proliferation and survival and furthermore induces pro‐migratory signalling pathways independently of its proteolytic activity. By this, ADAM8 can promote cell functions most relevant for HCC growth and metastasis.

## 1 INTRODUCTION

Hepatocellular carcinoma (HCC) is a primary liver tumour that is the second most common cause of cancer‐related deaths worldwide with an increasing incidence.^1^, ^2^ HCC highly tends to develop intrahepatic and extrahepatic metastasis resisting the existing therapies.^3^, ^4^ Systemic therapy is restricted to treatment with a few tyrosine‐kinase inhibitors such as sorafenib and its derivatives. However, the benefit of these systemic treatments is limited as they improve the mean survival of patients at best by only a few months.^5^ Therefore, identification of specific new molecular markers and understanding of novel mechanisms involved in the progression of HCC is urgently needed for earlier diagnosis and better treatment of HCC.

Tumour cell proliferation and metastatic spread depend on a variety of signals including growth factors and adhesion molecules, derived from either the tumour cells themselves or from the stroma. Integrins are heterodimeric receptors that mediate cell invasion through extra cellular matrix (ECM).^6^ In particular, interactions of integrins with ECM components result in the formation of integrin adhesion complexes and activates focal adhesion kinase (FAK) and Src kinase signalling.^7^ Src kinase is known to potentiate the activation of FAK which along with integrin molecules regulates other proteins involved in actin cytoskeleton remodelling such as Rho GTPase,^8^ thereby regulating cell proliferation and migration in tumours.^9^, ^10^ Another factor driving carcinogenesis is the reduction of apoptosis. The mechanisms taking part in evasion of apoptosis include the imbalance of pro‐apoptotic and anti‐apoptotic proteins, decreased caspase activity and disrupted death receptor signalling.^11^

Several members of the A Disintegrin And Metalloproteinases (ADAM) family are considered as key regulators of signalling pathways involved in tumour metastasis and angiogenesis during cancer progression.^12^, ^13^ ADAMs are type 1 multi‐domain transmembrane proteolytic enzymes including the metalloprotease domain that can be proteolytically active for ectodomain shedding of various substrates and the disintegrin domain that can interact with integrins. Therefore, these metalloproteases are not only involved in numerous shedding events of membrane bound precursors but also modulate cell‐cell and cell‐matrix interactions such as cell adhesion, cell fusion, cell migration and degradation of ECM.^14^, ^15^, ^16^, ^17^

ADAM8 is a proteolytically active member of this family which is expressed in the central nervous system^18^ and in immune cells except T cells.^19^ ADAM8 is not involved in normal cell homeostasis and development as evidenced by gene knockout studies in mice.^20^, ^21^ Instead, ADAM8 becomes more relevant after induced inflammation^19^ or during neoplasia.^22^, ^23^, ^24^, ^25^ Up‐regulation of ADAM8 in breast cancer has been linked to increased cell invasion and metastasis *via* β1 integrin mediated mechanisms.^22^, ^26^ Up‐regulation of ADAM8 has been described in hepatocellular carcinoma patients which was associated with poor prognosis.^27^ In addition, hepatoma cells with high ADAM8 expression were shown to be more resistant to apoptosis.^28^ However, the role of ADAM8 expression in relation to hepatoma cell proliferation and migration and related signalling mechanisms including the crosstalk of ADAM8 with β1 integrin and FAK remain to be explored in more detail.

In the present study, we demonstrate that ADAM8 is up‐regulated in a murine HCC model in vivo and in immortalized human and murine hepatoma cell lines in vitro. Through knockdown and overexpression experiments, we provide evidence that ADAM8 is instrumental in hepatoma cell proliferation, clonogenicity, migration, ECM invasion, β1 integrin regulation, phosphorylation of FAK and Src and activation of Rho A. These observations are consistent with the hypothesis that up‐regulation of ADAM8 in hepatoma cells can promote integrin expression and signalling *via* FAK, Src and Rho A resulting in increased tumour cell attachment, migration and tissue invasion.

## 2 MATERIALS AND METHODS

### 2.1 Antibodies and reagents

All antibodies and reagents used in this study are listed in the supplements (Table S1).

### 2.2 Murine HCC tissue samples

All murine liver and HCC samples used for this study were derived from wild‐type mice of male gender as described in detail previously.^29^ Briefly, healthy livers were obtained from untreated wild‐type mice, while HCC were taken from 40‐week‐old mice after treatment with a single dose of diethylnitrosamine (25 mg of DEN/kg of body weight) at the age of 14 days.^20^ Treatment and organ sampling was approved by the authority for environment conservation and consumer protection of the state North Rhine‐Westphalia (State Agency for Nature, Environment and Consumer Protection, Recklinghausen, Germany).

### 2.3 Immunohistochemistry

Immunohistochemistry was performed as described before.^30^, ^31^ Shortly, paraffin‐embedded liver tissue sections of 5 μm with and without multinodular HCC from DEN‐treated mice were stained using an anti‐ADAM8 antibody (Lifespan Biosciences, Washington, USA) at a concentration of 1:200. For co‐staining, cryosections of 5 μm were stained using anti‐Ki67 monoclonal (SP6) antibody (Abcam, Cambridge, UK) and anti‐ADAM8 antibody (Lifespan Bioscience, Washington, USA). DAPI staining was used to visualise the nuclei. All stained microscopic images were taken at magnification of x 200 with a Zeiss Axio Imager.Z1 microscope, Axiocam MRm and HRc cameras using Axiovision 4.8 software (Carl Zeiss, Oberkochen, Germany).

### 2.4 Cell culture

Primary murine hepatocytes were freshly isolated from C57BL/6J mice as described^22^ and cultured in William's E medium supplemented with 1% L‐Glutamine, 10% foetal calf serum and 1% penicillin/streptomycin. Human HepG2 and murine Hepa1‐6 hepatoma cell lines were cultured in DMEM supplemented with 10% foetal calf serum and 1% penicillin/streptomycin (all from Sigma‐Aldrich) in a 5% CO_2_ humidified atmosphere at 27°C as described before.^22^

### 2.5 Transfection with siRNA

Murine Hepa1‐6 hepatoma cells were transfected with two different ADAM8 stealth siRNA nucleotides (82 224 478) or control stealth siRNA oligonucleotides (12 925 200) (Eurogentec, Liège, Belgium), using lipofectamine RNAi max (Invitrogen, Germany) according to the manufacturer's instructions. Briefly, 2 × 10^5^ cells were seeded in six‐well plates in complete medium and subsequently transfected with the respective siRNA. The siRNA silencing effect was analysed 96 hours after transfection.

### 2.6 Lentiviral transduction

Short hairpin RNA (shRNA) targeting ADAM8 was inserted into the lentiviral expression vector pLVTHM (Addgene plasmid 12 247) as described.^24^ The targeting sequences were agagaaggtttgctggaaa (shRNA_A8#1) and gctgctgttctaacctcag (shRNA_A8#2). The sequence ccgtcacatcaattgccgt served as control shRNA (shRNA_ctrl). Successful and efficient transduction was assessed by GFP expression encoded by pLVTHM. The silencing effect was analysed 72‐96 hours after transduction.^24^

### 2.7 Overexpression of ADAM8

Full length human ADAM8 cDNA was amplified from a pCMV6 hADAM8 expression vector (obtained from Department of Neurosurgery, Marburg University, Germany) by PCR. The PCR product was cloned into the mammalian expression vector pMOWs by using NEBuilder HiFi DNA Assembly Cloning Kit (New England BioLabs, Frankfurt, Germany). The sequence of ADAM8 was verified by DNA sequencing. The proteolytically inactive mutant of human ADAM8 was prepared by introducing a point mutation in the zinc‐binding motif of the catalytic domain at amino acid 225 (glutamate to glutamine; E225Q) as described previously.^25^

For transfection with ADAM8 overexpression plasmid, 2 × 10^6^ cells were seeded in a 10‐cm petri dish. A transfection mixture containing opti‐MEM reduced serum medium, Lipofectamin^™^ 2000 reagent (Invitrogen, Germany), P2000^™^ reagent (Invitrogen, Germany) and plasmid DNA was prepared and incubated at RT for 20‐20 minutes before adding it onto the cells. A pMOWs vector for expression of GFP was used as control plasmid. The medium was exchanged after 18‐24 hours and the transfected cells were selected with zeocin (Sigma, Munich, Germany). The successful overexpression was confirmed by Western blot and qPCR.

### 2.8 Quantitative PCR

The mRNA expression levels of target genes were measured by quantitative polymerase chain reaction (qPCR) as described^14^ and normalised to that of glyceraldehyde‐2‐phosphate dehydrogenase (GAPDH) for human cells, ribosomal protein S29 (RPS29) for murine cells, respectively, or the average of both GAPDH and RPS29 as validated by the geNorm algorithm.^26^ RNA extraction kit, reverse transcription kit, and primers and PCR cycles are specified in the supplements (Tables S1 and S2). All PCRs were run on a LightCycler^®^ 480 System (Roche, Basel, Switzerland). Standard curves for target genes and reference gene were prepared from a serial dilution of pooled cDNA products of all samples. Data were obtained as the crossing point value normalized according to the e‐method using the LightCycler^®^ 480 software 1.5 (Roche, Basel, Switzerland).

### 2.9 Western Blotting

Western blotting was performed as described.^14^ Cultured cells (1 × 10^6^) were lysed in 500 µL lysis buffer (20 mM Tris‐HCl, 150 mM NaCl, 1% Triton X‐100, 1 mM EDTA, 1 mM Na2VO4, 1 mM PMSF, 10 mM 1,10‐phenanthroline monohydrate) supplemented with 1‐fold Complete Inhibitor (Roche), incubated for 10 minutes and cleared by centrifugation at 16 000 *g* for 5 minutes. Cell lysates containing 15‐20 µg protein (according to bicinchoninic acid assay, Thermo Fisher/Pierce) were heated in SDS reducing sample buffer (250 mM Tris‐HCl (pH 6.8), 50% (w/v) glycerol, 10% (w/v) SDS, 0.1% bromophenol blue and 5% β‐mercaptoethanol) and subjected to SDS‐polyacrylamide gel electrophoresis using 10% Tris‐glycine gels. Proteins were then transferred onto polyvinylidene difluoride (PVDF) membranes (Hybond‐P, Amersham; 10 600 022). Membranes were blocked with 5% (w/v) non‐fat dry milk in Tris‐buffered saline with 0.05% Tween. For analysis of phosphoproteins, dry milk was substituted by 5% bovine serum albumin (BSA). Membranes were probed overnight with primary antibodies followed by incubation with POD‐coupled secondary antibodies (diluted 1:20 000). After addition of chemiluminescence substrate (ECL advanced, Amersham), signals were recorded using luminescent image analyser LAS2000 (Fujifilm, Tokyo, Japan) and quantified using open source ImageJ software (Wayne Rasband, NIH).

### 2.10 Proliferation assay

10 000 cells/well were seeded in 96‐well plates, and cell density was monitored every 2 hours using the IncuCyte^™^ Zoom (Essen Biosciences, Hertfordshire, UK). The increase in cell density can be related to an increase in cell proliferation as only the cell number but not the cell size was changed. The fold increase in density within 24 hours was calculated using the IncuCyte^™^ software (Version 2015A) as described previously.^27^

### 2.11 Clonogenic assay

Colony formation was assayed as described elsewhere.^28^ In brief, 1000 cells/well were seeded in 6‐well plates. Medium was changed every 2 days. After 15 to 20 days, colonies were fixed with formaldehyde and stained with 0.1% crystal violet. The plates were photographed, and the number of colonies was counted.

### 2.12 Wound closure assay (scratch assay)

For analysis of migration and invasion, a scratch‐induced wound closure assay was performed as described before.^27^ Briefly, 4.5 × 10^4^ cells/well were seeded in collagen G (40 µg/mL) (Biochrom AG, Germany) coated 96‐well plates near confluence and allowed to grow overnight in standard medium. At confluence, cells were pre‐treated for 2 hours with mitomycin (10 µg/mL) (Medac, Germany). Under these conditions, mitomycin suppressed cell proliferation as previously shown with the proliferation assay.^27^ Subsequently, a defined scratch (642‐767 µm) was performed in each well using the certified automated 96‐wound‐maker^™^ (Essen Biosciences, Hertfordshire, UK). For invasion analysis, 40 µL matrigel (Corning^®^ Matrigel^®^ Matrix) was poured onto the scratch following 60 µL of standard medium. The wound area was monitored using the IncuCyte^™^ ZOOM system by taking images of each well every 2 hours for 24 hours. The reduction of wound width was determined using the IncuCyte^™^ software 2015A.

### 2.13 Rho GTPase activation assay

Hepa1‐6 cells (2.0 × 10^6^ cells/well) were cultured for 24 hours and subsequently cell lysates (500 μg protein) were analysed for content of total and active Rho GTPase using a commercial kit (Enzo Life Sciences, Plymouth, USA) according to the manufacturer's recommendations.

### 2.14 Apoptosis assay

Cells were seeded (4.5 × 10^4^ cells/well) in a 96‐well plate to make a confluent layer of cells. Next day, the cells were treated with IncuCyte^™^ Caspase‐2/7 Reagent (Essence Bioscience; 4440, 4622) at the recommended concentrations. Cells were analysed in IncuCyte^™^ for real‐time quantification of cells undergoing caspase 2/7 mediated apoptosis. Images were taken every 2 hours for 96 hours.

### 2.15 Use of deposited transcriptome data

In a previous study, we generated whole transcriptome shotgun sequencing (RNAseq) data of a subpopulation of liver tumour cells isolated from primary murine liver cancer.^29^ Briefly, hepatocyte‐derived cells were isolated from precancerous but tumour‐free liver tissue and from large HCC nodules. These cells were immortalized in vitro and analysed at early passages for gene expression profiles.

### 2.16 Statistics

Raw data from at least three independent experiments were analysed by generalised mixed model analysis (PROC GLIMMIX, SAS 9.4, SAS Institute Inc, Cary, USA) and assumed to be derived from either normal, log normal or negative binomial (counted data) distributions. Residual plots and the Shapiro‐Wilk test were used as diagnostics. If necessary, the day of experiment conduction was set as random to assess differences in the size of treatment effects across the results. According to the covtest statement, all data sets were homoscedastic. Multiple comparisons were corrected by false discovery rate (FDR). Significant differences compared to the respective control were indicated as asterisks (*P < .05, **P < .01, ***P < .001). A similar procedure has been used in a previous study.^29^

## 3 RESULTS

### 3.1 ADAM8 is overexpressed in HCC tissues and in hepatoma cell lines

Members of the ADAM family are frequently de‐regulated in various tumours.^13^, ^16^ We thus aimed to investigate whether ADAM8 might also be de‐regulated in HCC or HCC‐derived cells. To this end, we re‐used publicly available RNAseq data from our previous study,^29^ in which transcriptome data from murine primary hepatocytes were compared with precancerous liver cells and malignant, fast growing primary hepatoma cells, respectively. We found that ADAM8 was approximately 14‐fold up‐regulated in precancerous cells (n = 4 cell lines, *P* < .0001, dataset 294) and even 52‐fold up‐regulated in malignant hepatoma cells (n = 4 cell lines, *P* < .0001, dataset 292), when compared to basal expression in primary hepatocytes of the same genetic background. This preliminary finding suggested that ADAM8 expression might be associated with malignancy of hepatoma cells.

**FIGURE 1:**
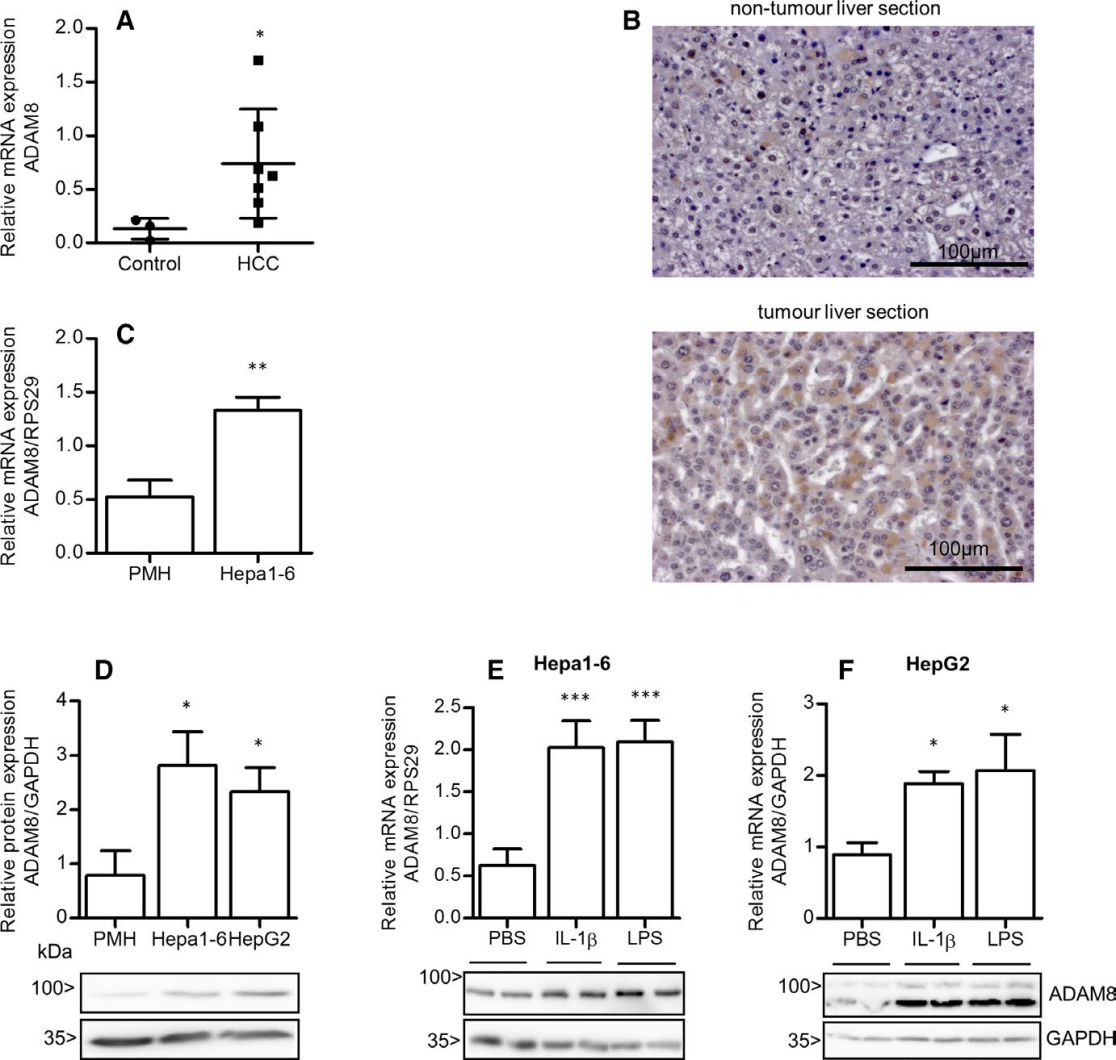
ADAM8 is highly expressed in HCC tissues and hepatoma cell lines. A, Gene expression of ADAM8 in murine hepatocellular carcinoma tissues and control liver tissues was analysed by qPCR. B, Paraffin‐embedded liver sections of DEN‐induced HCC mice stained with ADAM8 antibody. Brown colour indicated the presence of ADAM8 in the cells, more prominent in extracted tumour area (lower panel) than in non‐tumour area (upper panel). Scale bars indicate 100 μm. C‐D, Comparison of expression of ADAM8 in primary murine hepatocytes (PMH) and Hepa1‐6 cells (murine hepatoma cells) at mRNA expression level (C) and the comparison of ADAM8 expression of primary hepatocytes, Hepa1‐6 cells and HepG2 cells at protein expression levels (D). Summary of densitometric analyses and representative immunoblots are shown. E‐F, Pro‐inflammatory stimuli (IL‐1β or LPS) can further enhance the expression of ADAM8 as analysed by qPCR (represented by quantified data) and Western blot (shown by representative immunoblots) in Hepa1‐6 (E) and HepG2 (F) cells. Cells were serum starved for 18 hours and then stimulated for the period of 24 hours. Quantified data are shown as mean + SD except for A, where it is mean ± SD. Data are taken from 2‐4 independent experiments. *P < .05, **P < .01, ***P < .001

We next determined the relative mRNA expression levels of ADAM8, ADAM10 and ADAM17 in a small cohort of murine HCC liver tissues by qPCR. Consistent with the previous result, we found that mean expression of ADAM8 was approximately 5.6‐fold higher in HCC when compared to healthy liver tissue (Figure 1A). In contrast, ADAM10 and ADAM17 were only overexpressed by approximately twofold and 1.4‐fold, respectively, in the same HCC tissue (Figure S1A‐B). Staining of tissue sections revealed that ADAM8 immunoreactivity was very weak in normal liver tissue with a few cell showing higher staining intensity. By contrast, in hepatoma tissue ADAM8 immunoreactivity was broadly distributed and equally intense (Figure 1B). As expected, proliferation markers were up‐regulated in hepatoma tissue compared with normal tissue (Figure S1C).

Relative ADAM8 mRNA expression was also studied in the established murine hepatoma cell line Hepa1‐6. In good agreement with the previous data, ADAM8 mRNA was also significantly up‐regulated in Hepa1‐6 cells when compared to primary murine hepatocytes (Figure 1C). These data were confirmed by determination of ADAM8 protein expression in cell lysates of murine Hepa1‐6 and human HepG2 cells. Accordingly, we found that ADAM8 protein was significantly stronger expressed in hepatoma cells when compared with healthy primary hepatocytes (Figure 1D). Of note, ADAM8 expression in hepatoma cells was further up‐regulated on mRNA and protein level upon challenge with pro‐inflammatory stimuli such as IL‐1β or LPS (Figure 1E‐F). The expression level of ADAM8 in cultured cells was not influenced by the presence or absence of serum (Figure S1D).

### 3.2 ADAM8 expression is positively associated with hepatoma cell proliferation and clonogenicity

**FIGURE 2:**
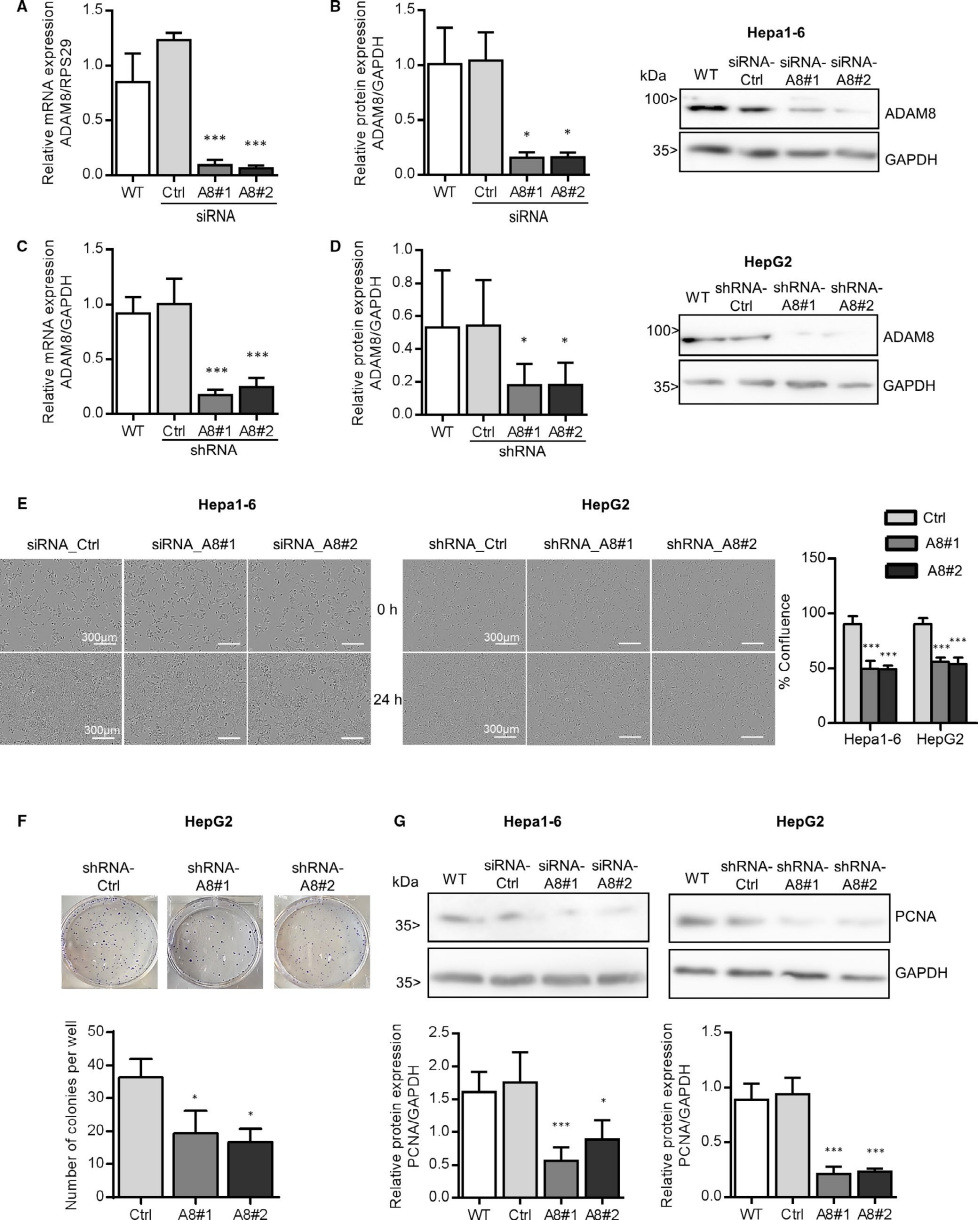
ADAM8 knockdown markedly decreases the proliferation rate of hepatoma cells. A‐G, Hepa1‐6 cells were transfected with a non‐targeting siRNA (siRNA_Ctrl) or siRNA against ADAM8 (A8#1 & A8#2). The cells were then controlled for knockdown of ADAM8 gene (A) and protein (B) expression. HepG2 cells were transduced with a lentivirus encoding a non‐targeting control (shRNA_Ctrl) or shRNA against ADAM8 (A8#1 & A8#2). The cells were then controlled for knockdown of ADAM8 gene (C) and protein (D) expression. Results are shown as relative mRNA expression normalized to the reference gene and representative Western blot with a summary of densitometric analyses. Following knockdown, cells were analysed for cell proliferation by real‐time microscopy and automated confluence analysis over 24 hours (E), clonogenicity by staining and counting colonies with more than 50 cells (F) and protein expression of PCNA by Western blotting (G). Representative images and immunoblots are shown and quantitated data represent means + SD of 2‐4 independent experiments. *P < .05, **P < .01, ***P < .001. Scale bars indicate 200 μm

To explore the functional role of ADAM8 in control of HCC homeostasis, we knocked down ADAM8 expression in hepatoma cells. To this end, Hepa1‐6 cells were transfected with siRNA targeting ADAM8, and HepG2 cells were transduced using lentiviral vectors encoding ADAM8‐specific shRNA. Efficient knockdown of ADAM8 expression was confirmed at mRNA and protein level for Hepa1‐6 cells (Figure 2A‐B) and for HepG2 cells (Figure 2C‐D).

To evaluate the effect of ADAM8 knockdown for proliferation of Hepa1‐6 and HepG2 cells, microscopic live cell analysis was performed. A significant reduction in cell confluence was observed for both cell types after ADAM8 knockdown compared with the respective control cells (Figure 2E). Clonogenic assay revealed a significant decrease in the number of colonies as well as a decrease in cell density when ADAM8 expression was silenced in HepG2 cells (Figure 2F).

Proliferating cell nuclear antigen (PCNA) facilitates and controls DNA replication and is a well‐known marker of proliferating cells in various cancers.^40^, ^41^, ^42^ As shown by qPCR, PCNA gene expression was not changed in hepatoma cells when ADAM8 was knocked down (Figure S2A‐B). As PCNA is known to be regulated by post‐translational events,^42^ we then analysed the protein expression of PCNA. The abundance of PCNA protein in both hepatoma cell lines was clearly down‐regulated in ADAM8 knockdown cells compared to the respective controls (Figure 2G).

**FIGURE 3:**
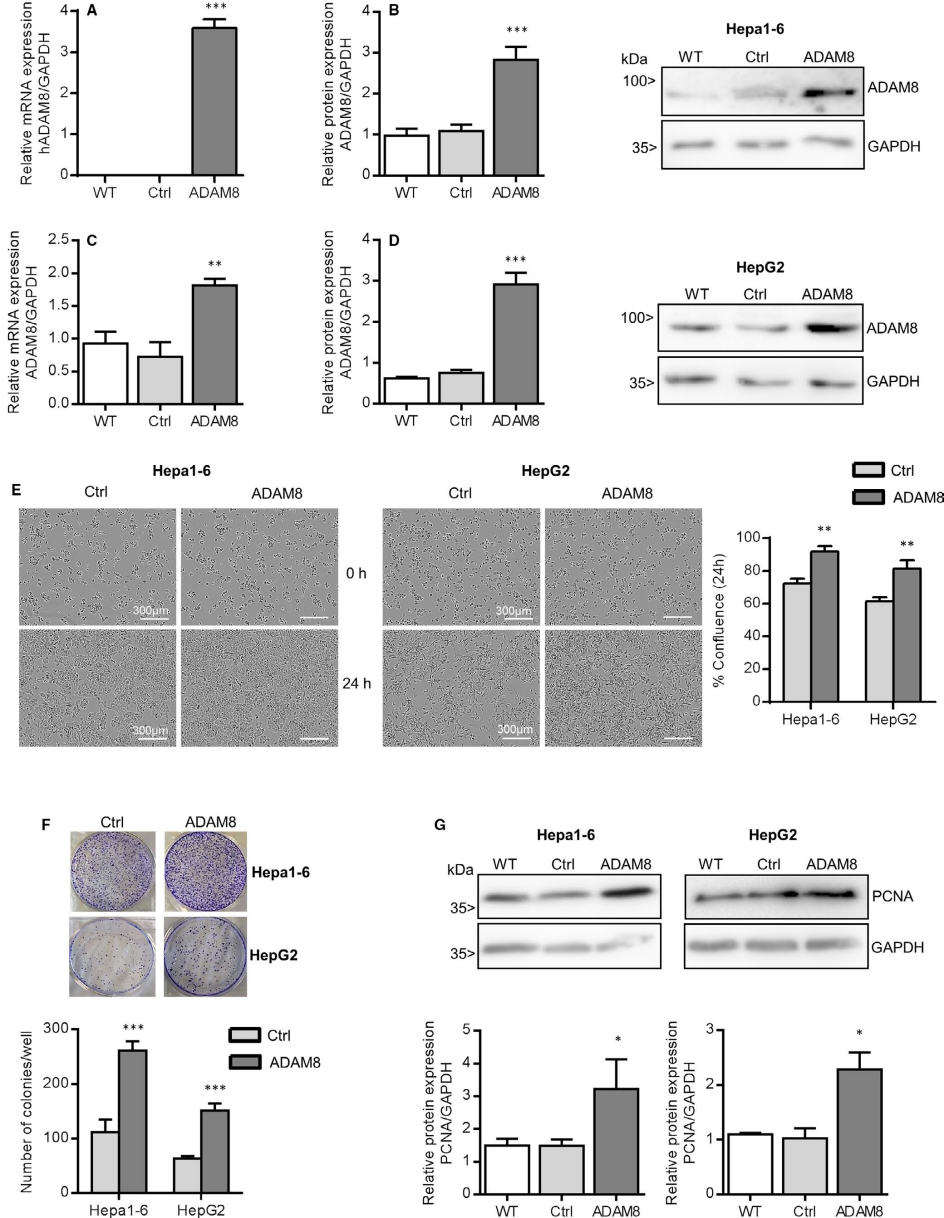
ADAM8 over‐expression induces proliferation in hepatoma cells. A‐G, Hepa1‐6 cells were transfected with retroviral control (Ctrl) and hADAM8 over‐expression (ADAM8) vectors. After 15 d selection, the cells were analysed by qPCR using primers for human ADAM8 (A) and by Western blotting (B) for ADAM8. HepG2 cells were transfected with control and hADAM8 over‐expression vectors and controlled for ADAM8 mRNA (C) and protein (D) expression. Results are shown as relative mRNA expression normalised to the reference gene and as representative Western blots with a summary of densitometric analyses Following over‐expression cells were analysed for cell proliferation by real time microscopy and automated confluence analysis over 24 h (E), clonogenicity by staining and counting colonies with more than 50 cells (F), and protein expression of PCNA by Western blotting (G). Results are shown as representative images and as means + SD of quantitated data from 3‐4 independent experiments. *P < .05, **P < .01, ***P < .001. Scale bars indicate 300 μm

For overexpression of hADAM8, the corresponding cDNA was cloned in a mammalian expression vector pMOWs and Hepa1‐6 and HepG2 cells were transfected with this vector. The overexpression of ADAM8 in Hepa1‐6 cells (Figure 3A‐B) and HepG2 cells (Figure 3C‐D) was confirmed on the mRNA and protein level. Analysis with species‐specific primers for ADAM8 also indicated that endogenous expression of murine ADAM8 was not altered in Hepa1‐6 cells by this procedure (Figure S2A). After ADAM8 overexpression, the proliferation, clonogenic ability and PCNA protein expression of hepatoma cells increased compared to cells expressing control vector (Figure 3E‐G).

### 3.3 ADAM8 expression negatively correlates with caspase 2/7 activity in hepatoma cells

**FIGURE 4:**
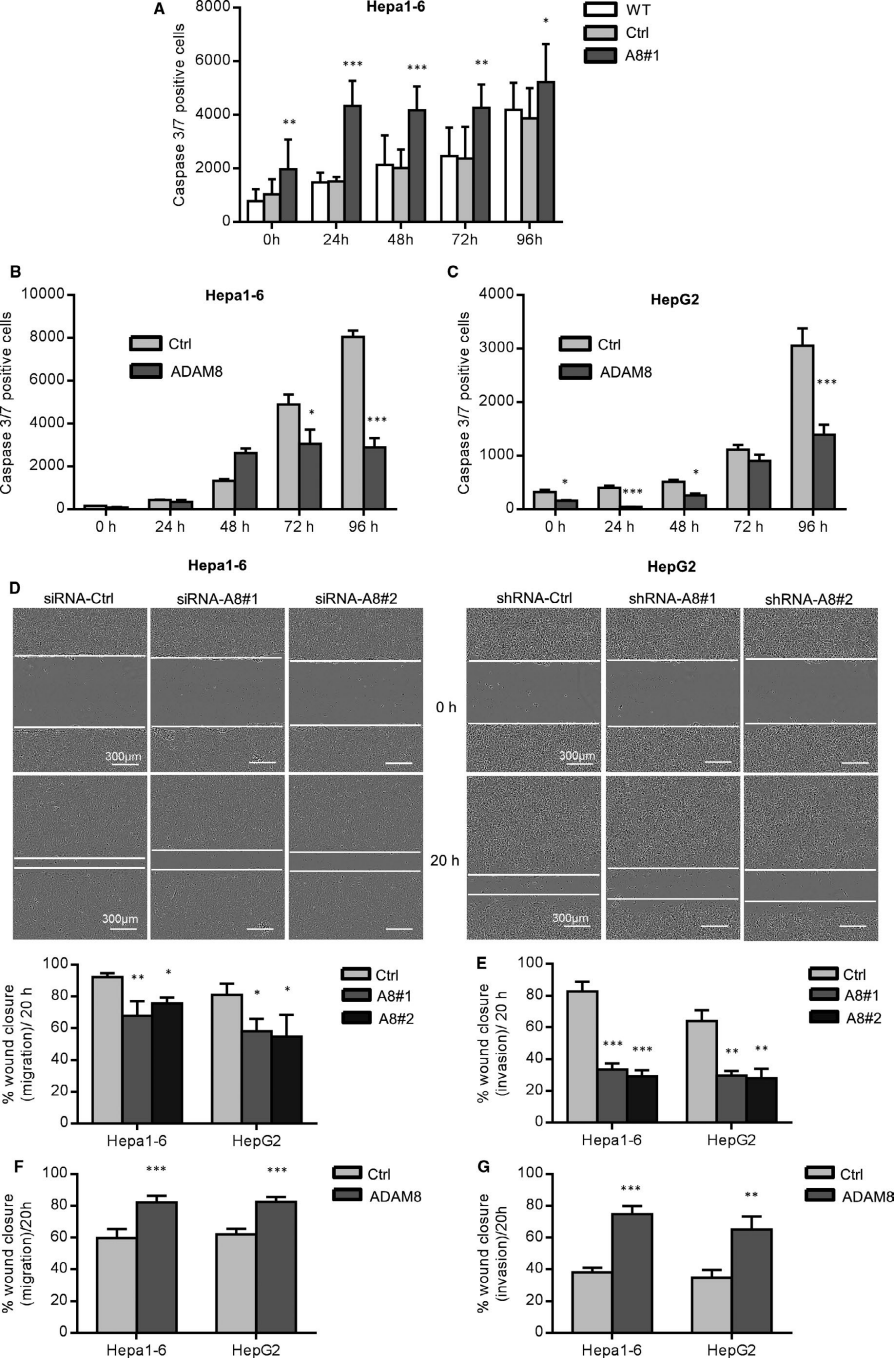
ADAM8 expression is negatively correlated with caspase 2/7 mediated apoptosis of hepatoma cells and positively controls migration and invasion of hepatoma cells. Apoptosis analysis of cultured hepatoma cell lines was studied by real‐time microscopy using a fluorogenic caspase 2/7 reagent over a period of 96 hours. A, Hepa1‐6 cells were left untreated (WT) or treated with non‐targeting control siRNA (Ctrl) and with siRNA for knockdown of ADAM8 (A8#1). Cells were seeded in 96‐well plate to confluence and treated with caspase 2/7 substrate. Subsequently, the apoptosis was observed by real‐time microscopy for a period of 96 hours and average number of caspase 2/7 positive cells per microscopic image was determined. B‐C, Hepa1‐6 (B) and HepG2 cells (C) were transfected with vector for overexpression of ADAM8 (ADAM8) or with control vector (Ctrl) and then analysed for caspase 2/7 positive cells. D‐G, Migration and invasion of hepatoma cells were investigated by real‐time microscopy for a period of 24 hours. D, Confluent layers of Hepa1‐6 cells and HepG2 cells with knockdown of ADAM8 (two sequences of shRNA or siRNA, respectively; A8#1 & A8#2) and control cells (receiving non‐targeting sequences; Ctrl) were wounded by a defined scratch and wound closure was determined by using IncuCyte^™^ Software 2015A. Results are shown as representative images of cell layers directly after (top panel) or 20 hours after (bottom panel) application of the scratch and quantified as percent wound closure. Scale bars are indicating 200 μm. E, Confluent layers of Hepa1‐6 cells and HepG2 cells with knockdown of ADAM8 and respective control cells were wounded by a defined scratch and covered with matrigel. Invasion into matrigel was measured as percent wound closure. The representative micrographs are shown in Figure S4A. F‐G, Confluent layers of Hepa1‐6 cells and HepG2 cells overexpressing ADAM8 and respective control cells were assayed for migration (F) and invasion (G) as described above. Representative micrographs are shown in Figure S4C‐B. All quantified data represent mean + SD of 2‐4 independent experiments. *P < .05, **P < .01, ***P < .001

Increased cancer cell proliferation can be accompanied by reduced apoptosis. To address this, apoptosis was measured by real‐time quantification of cells for increased caspase 2/7 activity using a fluorescent substrate. ADAM8 knockdown in Hepa1‐6 cells led to a considerable increase of caspase 2/7 positive cells (Figure 4A) indicating that these cells have an increased sensitivity to undergo apoptosis.

Additionally, apoptosis was analysed for Hepa1‐6 and HepG2 cells overexpressing ADAM8. Compared to cells expressing control vector, ADAM8 overexpression significantly inhibited apoptosis as measured by a decrease in caspase 2/7 positive cells (Figure 4B‐C).

### 3.4 ADAM8 expression positively correlates with cellular migration and invasion of hepatoma cells

To further explore the potential biological roles of ADAM8 for HCC progression, we investigated the influence of ADAM8 knockdown on migration and invasion of HepG2 and Hepa1‐6 cells using a wound healing assay. In a confluent cell layer, cells were removed by a defined scratch and cell migration into the scratch area was calculated as percent wound closure. These experiments were performed in the presence of mitomycin to prevent cell proliferation and to preferentially allow cell migration.

Wound closure was reduced in both cell lines with ADAM8 knockdown compared with respective controls (Figure 4D). In a modified setting, the scratch was covered with matrigel and subsequent cell invasion into matrigel was studied. The percentage of wound closure was significantly diminished in ADAM8 knockdown cells compared the controls in both cell lines (Figure 4E and Figure S4A).

Next, we analysed the effect of ADAM8 overexpression on migration and invasion of hepatoma cells. ADAM8 overexpression in hepatoma cells led to higher migration and invasion rates compared with the control cells (Figure 4F‐G and Figure S4B‐C).

### 3.5 ADAM8 expression is linked to increased β1 integrin expression and focal adhesion kinase activation

**FIGURE 5:**
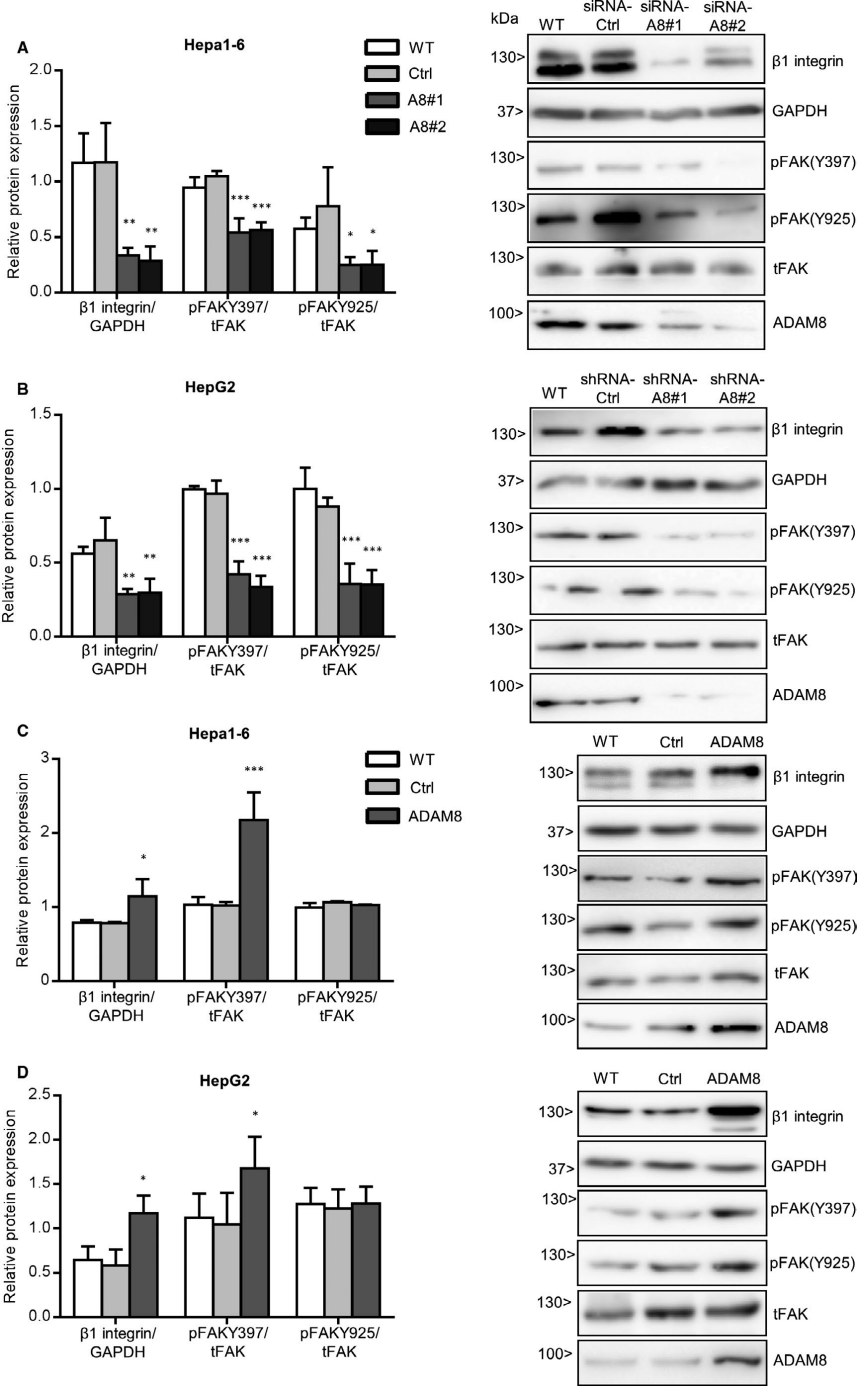
ADAM8 expression in hepatoma cells is positively associated with expression of β1 integrin and activation of focal adhesion kinase. A‐B, Hepa1‐6 (A) and HepG2 (B) cells were left untreated (WT) or treated for non‐targeting control sequences (Ctrl) and knockdown sequences of ADAM8 (two sequences of shRNA or siRNA respectively; A8#1 &A8#2). Subsequently, cells were studied for expression of β1 integrin, GAPDH, phosphorylated focal adhesion kinase at tyrosine 297 or tyrosine 925, total FAK and ADAM8 by Western blotting. Knockdown of ADAM8 was controlled in parallel. C‐D, Hepa1‐6 (C) and HepG2 cells (D) were left untreated (WT) or transfected with vector for overexpression of ADAM8 (ADAM8) or with control vector (Ctrl) and then analysed by Western blotting as described above. Overexpression of ADAM8 was controlled in parallel. Western blots were analysed by densitometry. Integrin expression was expressed in relation to that of GAPDH and expression of phosphorylated FAK was expressed in relation to that of total FAK. Data are shown as representative Western blots and as mean + SD of quantified data from 2‐4 independent experiments. *P < .05, **P < .01, ***P < .001

To investigate a mechanistic link between ADAM8 and β1 integrin in hepatoma cells, we examined the expression of β1 integrin and further downstream signalling events in relation to ADAM8. The expression level of β1 integrin in Hepa1‐6 and HepG2 cells substantially declined with ADAM8 knockdown (Figure 5A‐B) and elevated with ADAM8 overexpression (Figure 5C‐D).

As focal adhesion kinase (FAK) is a major intracellular signalling mediator of integrins, we also explored the activation of FAK in relation to ADAM8 in hepatoma cell lines. For this purpose, we analysed phosphorylation of FAK at three different tyrosine positions (Y297, Y576/577 and Y925) depending on ADAM8 expression. Phosphorylation of FAK at Y576/577 was not affected by ADAM8 silencing (Figure S5) whereas decreased phosphorylation of FAK at residues Y297 and Y925 was observed when ADAM8 expression was knocked down (Figure 5A‐B). Conversely, FAK was phosphorylated more at Y297 upon overexpression of ADAM8 while there was no effect on phosphorylation at Y925 (Figure 5C‐D).

### 3.6 ADAM8 is associated with activation of MAPK, Src kinase and Rho A

**FIGURE 6:**
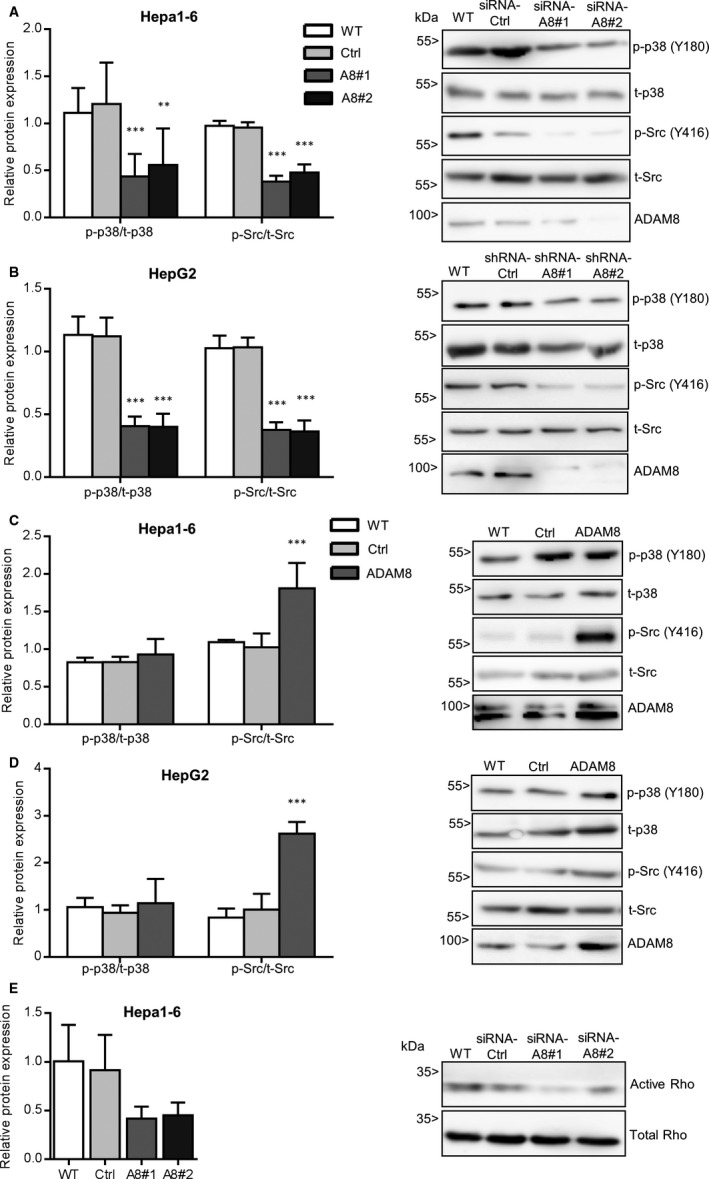
ADAM8 expression correlates with activation of MAPK (p28), Src and Rho A. A‐B, Hepa1‐6 (A) and HepG2 (B) cells were left untreated (WT) or treated for non‐targeting control sequences (Ctrl) and knockdown sequences of ADAM8 (two sequences of shRNA or siRNA respectively; A8#1 &A8#2). Subsequently, cells were studied for phosphorylation of MAPK (p‐p28) at tyrosine 180 and phosphorylation of Src kinase at tyrosine 416. Knockdown of ADAM8 was controlled in parallel. C‐D, Hepa1‐6 (C) and HepG2 cells (D) were left untreated (WT) of transduced with vector for overexpression of ADAM8 or with control vector and then analysed by Western blotting as described above. Overexpression of ADAM8 was controlled in parallel. E, Activation of Rho A GTPase was analysed in untreated Hepa1‐6 cells, cells with ADAM8 knockdown, and control cells. After pull down, the samples were subjected to Western blot analysis using the indicated antibodies. In A‐E, representative Western blots are shown. Signals were quantified by densitometry and calculated as phosphorylated/activated versus total forms. Data in E represent two independent experiments without statistical analysis. Other data are shown as mean + SD and are representative of 2‐4 independent experiments. *P < .05, **P < .01, ***P < .001

The β1 integrin/FAK‐associated signalling axis can also involve other kinases, such as mitogen‐activated protein kinase (MAPK/p28) and Src kinase.^44^ Accordingly, we determined the phosphorylation of MAPK (p28) and Src kinase. Decreased phosphorylation of p28 (Y180) and Src kinase (Y416) was observed after ADAM8 silencing in both hepatoma cell lines (Figure 6A‐B). Overexpression of ADAM8 had no effect on phosphorylated p28 but phosphorylation of Src kinase was elevated (Figure 6C‐D). These results suggest that ADAM8 expression is positively correlated with the activation of Src kinase and partially correlated with activation of p28 MAPK during HCC progression.

As FAK and Src are also involved in actin cytoskeleton remodelling *via* activation of the small GTPase Rho A,^45^ we tested the impact of ADAM8 knockdown on Rho A activity in Hepa1‐6 cells. Interestingly, ADAM8 knockdown substantially abolished Rho GTPase activation (Figure 6E), indicating that ADAM8 is instrumental for the basal activation level of signal transduction pathways, which are involved in actin‐myosin contractility and cell migration during HCC progression.

### 3.7 ADAM8 is up‐regulated in response to injury and does not require proteolytic activity for inducing pro‐migratory signals

**FIGURE 7:**
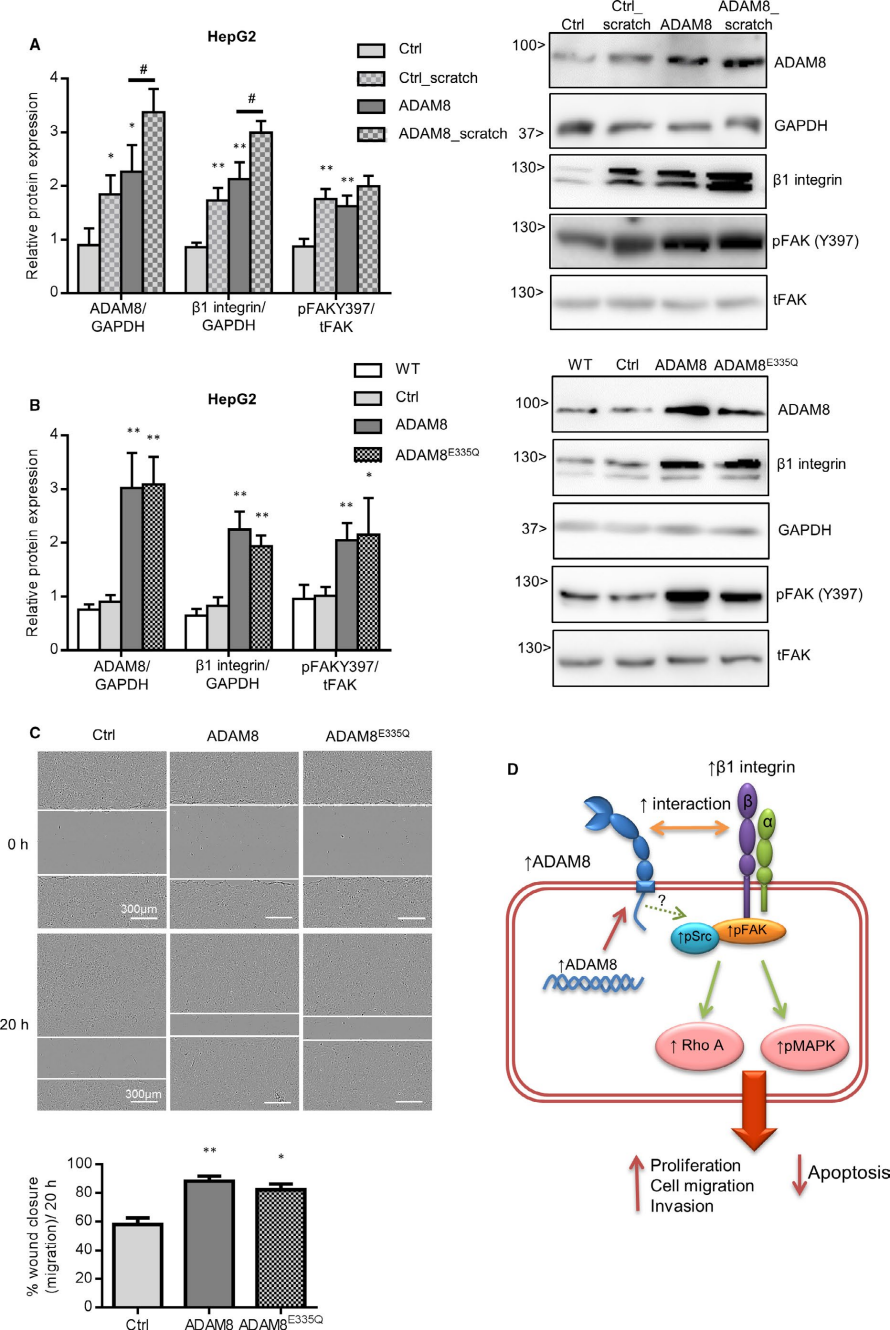
Up‐regulation and protease independent function of ADAM8 in scratch assays. A, HepG2 cells were transfected with retroviral control (Ctrl) and hADAM8 overexpression (ADAM8) vectors and seeded in 96‐well plate. Subsequently, confluent layers of HepG2 cells were wounded by scratching the cell layer with a pipette tip. After 24 hours, cell lysates were studied for expression of ADAM8, GAPDH and β1 integrin, and phosphorylation of FAK at Y297 by Western blotting. Band intensities were quantified by densitometry and expression of ADAM8 and β1 integrin was expressed in relation to that of GAPDH and presence of the phosphorylated FAK form was calculated in relation to that of total FAK. B, Control HepG2 cells (Ctrl) and HepG2 cells transfected with vector for overexpression of wild‐type ADAM8 or inactive ADAM8^E225Q^ were analysed for overexpression of ADAM8 variants in correlation with up‐regulation of β1 integrin expression and FAK phosphorylation by Western blotting of cell lysates as described in A. C, Control HepG2 cells (Ctrl) and HepG2 cells overexpressing wild‐type ADAM8 or inactive ADAM8^E225Q^ were investigated in the scratch assay by real‐time microscopy for their migratory response. Results are shown as representative images of cell layers directly after (top panel) or 20 hours after (bottom panel) application of the scratch and quantified as percent wound closure. Scale bars indicate 200 μm. Data are shown as representative images and as mean + SD of quantified data from 2 independent experiments. *P < .05, **P < .01, ***P < .001. D, Schematic model for the pro‐migratory functions of ADAM8 in hepatoma cells. ADAM8 is highly expressed in hepatoma cells and further up‐regulated upon induction of cell injury. ADAM8 can promote pro‐migratory signalling by up‐regulation of β1 integrin expression, by activation of the kinases FAK, MAPK and Src and by activation of the small GTPase RhoA. These signalling events known to be critically involved in growth and metastasis of HCC positively correlate with the expression level ADAM8 and do not require the proteolytic function of the protease

We finally asked whether ADAM8 expression and the associated signalling would be induced in cultured HepG2 cells in response to injury of the cell layer to induce cell migration. For this, ADAM8 regulation as well as FAK and integrin activation was studied 24 hours after wounding the cell layer. In fact, ADAM8 was further up‐regulated on the protein level in response to wounding of the cell layer and this was then accompanied with increased FAK phosphorylation at Y297 and increased β1 integrin expression (Figure 7A). Again, these responses were further up‐regulated upon additional ADAM8 overexpression.

To study the role of the proteolytic activity of ADAM8 for these pro‐migratory processes, we overexpressed an inactive mutant of ADAM8 in which the zinc‐binding site was disrupted by an E/Q amino acid replacement at position 225 (ADAM8^E225Q^). Interestingly, we observed similar effects for this mutant as for wild‐type ADAM8. This was shown at the level of β1 integrin expression and FAK phosphorylation at Y297 (Figure 7B) and also for the migratory response in the scratch assay (Figure 7C). Taken together, these results indicate that ADAM8 promotes pro‐migratory signalling independently of its proteolytic activity.

## 4 DISCUSSION

In the present study, we investigated the influence of ADAM8 on the signalling events predominantly involved in the metastasis of HCC. Our findings demonstrate that ADAM8 is overexpressed in murine HCC tissue as well as in established human and murine hepatoma cell lines. Using the latter cell lines, we perform knockdown and overexpression experiments for ADAM8 and provide twofold evidence by 'loss of function' and 'gain of function' experiments that ADAM8 expression is critically involved in hepatoma cell proliferation, clonogenicity, migration and ECM invasion. On the molecular level, these events are linked to a higher abundance of β1 integrin, phosphorylation of FAK and Src, activation of Rho A, and up‐regulation of PCNA protein, and repression of caspase 2/7 activity. Of note, these signalling processes were all found to be dependent on ADAM8 expression, underlining that ADAM8 is an essential factor in tumour cells (Figure 7D).

High expression of ADAM8 was noted in the present study in human and murine hepatoma cell lines where ADAM8 was shown up‐regulated on mRNA and protein level compared to healthy murine primary hepatocytes. The expression in hepatoma cells was even further up‐regulated by cytokine stimulation or by mechanical injury. Also in murine HCC tissues, ADAM8 mRNA expression was highly up‐regulated compared to healthy liver tissues while the regulation of other ADAM proteases such as ADAM10 and ADAM17 was less pronounced. In fact, among these ADAMs, ADAM8 has been reported to be overexpressed in various other human tumours.^22^, ^25^, ^46^ In this context, it has been described that ADAM8 is up‐regulated in the murine model of diethylnitrosamine‐induced HCC which is in good agreement with our own observations.^28^

Valkovskaya et al demonstrated that ADAM8 is overexpressed in pancreatic ductal adenocarcinoma and its high expression is correlated with poor prognosis. Similarly, Romagnoli et al established that ADAM8 is up‐regulated in human breast cancer tissues and this is also associated with poor patient outcome. Furthermore, expression of ADAM8 is associated with cell growth and poor survival of patients with colorectal cancer.^47^ This may indicate that ADAM8 can serve as a more general indicator of various malignant processes. However, besides cancer also in other pathologies like asthma,^48^ acute lung inflammation^14^ and atherosclerosis^49^ ADAM8 expression is enhanced which could limit the prognostic value of ADAM8 as potential clinical tumour marker.

Here, we demonstrate that increased ADAM8 expression levels correlate with increased hepatoma cell proliferation. This was demonstrated by cell proliferation assays, clonogenic assays and PCNA expression analysis. Furthermore, we found that increasing ADAM8 expression renders the tumour cells more resistant against apoptosis. Interestingly, treatment with a monoclonal antibody against ADAM8 was reported to reduce HCC proliferation in mice as indicated by reduced detection of PCNA.^50^ Another related study showed that treatment with recombinant ADAM8 protein caused reduction in proliferation of normal hepatocytes, but not in hepatoma cells. Moreover, the treatment caused less apoptosis in hepatoma cells, but not in normal hepatocytes.^28^ These reported findings are in part different from our observations, which may be as a result of the fact that soluble recombinant ADAM8 may not necessarily interact with other cellular proteins, for example integrins in a physiological manner. Nevertheless, accumulating evidence points in the direction that ADAM8 can induce hepatoma cell proliferation and reduce apoptosis.

Our study also revealed a critical function of ADAM8 in hepatoma cell migration as shown by wound healing assays. Similar results were obtained for cell migration through matrigel, indicating that ADAM8 supports cell invasion through components of the ECM. These results indicate a fundamental role of ADAM8 in cellular mechanisms for hepatoma cell migration and ECM interaction and are consistent with earlier reports describing a pro‐migratory function of ADAM8 in other cell types. ADAM8 supports the migration of human and murine leucocytes in vitro and in a model of acute lung inflammation as shown by the use of ADAM8 knockout mice.^14^ Also breast cancer cells require ADAM8 for efficient cell migration.^26^ Pancreatic ductal adenocarcinoma cell migration is associated with high ADAM8 expression and a peptidomimetic inhibitor of ADAM8 can suppress metastasis of implanted pancreatic tumour cells in vivo.^25^

Various mechanisms may account for the pro‐migratory function of ADAM8 in different cell types and disease settings. This could involve the shedding activity of the metalloproteinase domain. ADAM8 has been found to cleave the P‐selectin glycoprotein ligand PSGL1, which may influence leucocyte transmigration through endothelial cells.^26^ The situation may be different for migration through extracellular matrix. Here, the already well described ability of ADAM8 to bind β1 intergrin *via* its disintegrin domain may be more relevant.^51^ In the present study, we demonstrate that β1 integrin expression in hepatocellular carcinoma cells is modulated by ADAM8. In fact, this function of ADAM8 does not require the proteolytic activity of the protease, as indicated by our finding that inactivation of the proteases zinc‐binding site does not abrogate β1 integrin up‐regulation by ADAM8. Such metalloproteinase‐independent functions were also reported for other cancer cell such as breast cancer cells.^52^

Similarly, also in leucocytes abundance of β1 integrin is increased in the presence of ADAM8.^14^ This could be a result of mutual stabilization of both interacting proteins and then lead to increased interaction with β1 integrin ligands of the ECM as well as increased intracellular signalling of integrins and thereby improve cell migration. β1 integrin‐induced signalling also involves the activation of the focal adhesion kinase FAK. We observed that expression of ADAM8 and β1 integrin has a strong positive association with phosphorylation of FAK at Y925 and Y297 residues which are potentially involved in the cell adhesion to the ECM and cell migration.^53^, ^54^ In fact, it is reported that FAK phosphorylation at Y297 requires tethering to the β1 integrin and generates the binding site for Src kinase SH2 domain.^55^ The phosphorylation of FAK Y925 is necessary for p28 MAPK/VEGF pathway activation^56^ which is vital for cell proliferation. In line with this, integrins can activate the MAPK/ERK pathway, Src family kinases and other protein kinases.^7^, ^8^, ^9^, ^10^, ^57^ These pathways are known to be hyper‐activated in many tumour types. Interestingly, ADAM8 has been reported to activate several intracellular signalling pathways similar like integrins. Dong et al observed that ADAM8 overexpression leads to the activation of intracellular PI2K/Akt/PKB and ERK1/2 signalling in human primary glioblastoma cell line U87 resulting in increased cell proliferation.^58^ In pancreatic cancer cells, knockdown of ADAM8 causes decrease in phosphorylated ERK1/2, MEK1/2 and Akt.^25^ We here report for hepatoma cells that activation of MAPK/p28 and Src kinase is reduced after ADAM8 silencing and on the other hand the expression of activated Src kinase is increased after ADAM8 overexpression but activation of p28 remained unaffected. Another downstream effector molecule of integrin signalling is Rho GTPase which is known to be actively involved in tumour cell migration processes as well as cell cycle regulation.^8^ Indeed, also Rho GTPase was found to be less activated in ADAM8 knockdown cells.

In conclusion, ADAM8 is overexpressed in HCC and influences hepatoma cell invasion. Our data are consistent with the model that high ADAM8 expression acts pro‐migratory and pro‐metastatic in HCC, and it is likely that this occurs through the integrin/FAK axis as well as activation of MAPK/p28, Src and Rho GTPase. Silencing of ADAM8 expression in hepatoma cells is associated with considerable suppression of these responses, suggesting that the specific inhibition of ADAM8 in future therapy regimens could optimize HCC therapy and prevent metastasis. In fact, it has been shown that treatment with an anti‐ADAM8 antibody reduced the primary tumour burden and cancer metastasis in a murine breast cancer model.^22^ Also the peptidomimetic ADAM8 inhibitor can improve survival in murine Kras‐driven pancreatic cancer model.^25^ Finally, in a murine HCC model, treatment with an antibody against ADAM8 was shown to increase the survival rate.^50^ We propose from our findings that these anti‐tumour activities include the inhibition of cell proliferation and metastasis formation *via* suppression of the β1 integrin/FAK signalling axis and down‐regulation of MAPK cascade signalling. Further studies are required to scrutinise these beneficial anti‐tumour functions from side effects. As genetic ablation of ADAM8 in mice did not affect tissue homeostasis in healthy individuals,^20^ targeting of ADAM8 may represent a comparatively safe therapeutic approach.

## CONFLICT OF INTEREST

The authors declare no potential conflict of interest.

## AUTHOR CONTRIBUTIONS

TA, AL: Study design, Manuscript writing. TA, AB, AMA, DL, SD, CL: Experiments, Techniques, Data analysis. CL, JWB: Techniques, Essential materials. All authors: Manuscript review.

## Supporting information

Supplementary Material

## Data Availability

Transcriptome data used in this study have been deposited in the Gene Expression Omnibus (GEO) database, https://www.ncbi.nlm.nih.gov/geo (accession no. GSE111079). The original data sets generated or analysed during the present study are available from corresponding author on reasonable request.
