# Supplementary material for: Expression levels of the metalloproteinase ADAM8 critically regulate proliferation, migration and malignant signalling events in hepatoma cells
Source: J Cell Mol Med. 2020 Dec 13;25(4):1982–99. doi: 10.1111/jcmm.16015 (PMC7882935; doi:10.1111/jcmm.16015)
Supplement: Supplementary file 1 — Supplementary Material [file JCMM-25-1982-s001.pdf]

Expression levels of the metalloproteinase ADAM8 critically regulate proliferation, migration and malignant signalling events in hepatoma cells

Tanzeela Awan[1], Aaron Babendreyer[1], Abid Mahmood Alvi[1], Stefan Düsterhöft[1], Daniela Lambertz[3], Jörg W. Bartsch[2], Christian Liedtke[3], Andreas Ludwig[1*]

[1]Institute of Pharmacology and Toxicology, Medical Faculty, RWTH Aachen University, Aachen, Germany

[2]Department of Neurosurgery, Philipps University Marburg, University Hospital Marburg, Marburg, Germany

[3]Department of Medicine III, University Hospital RWTH Aachen University, Aachen, Germany

The authors declare no potential conflict of interest.

Running title: ADAM8 promotes malignant hepatoma cell functions

*Address correspondence to:

Andreas Ludwig,

Institute of Pharmacology and Toxicology,

RWTH Aachen University, Pauwelsstr. 30, 52074 Aachen, Germany;

Phone: +49 241 8035771, Fax: +49 241 8082433, E-Mail: aludwig@ukaachen.de

Supplementary Figures

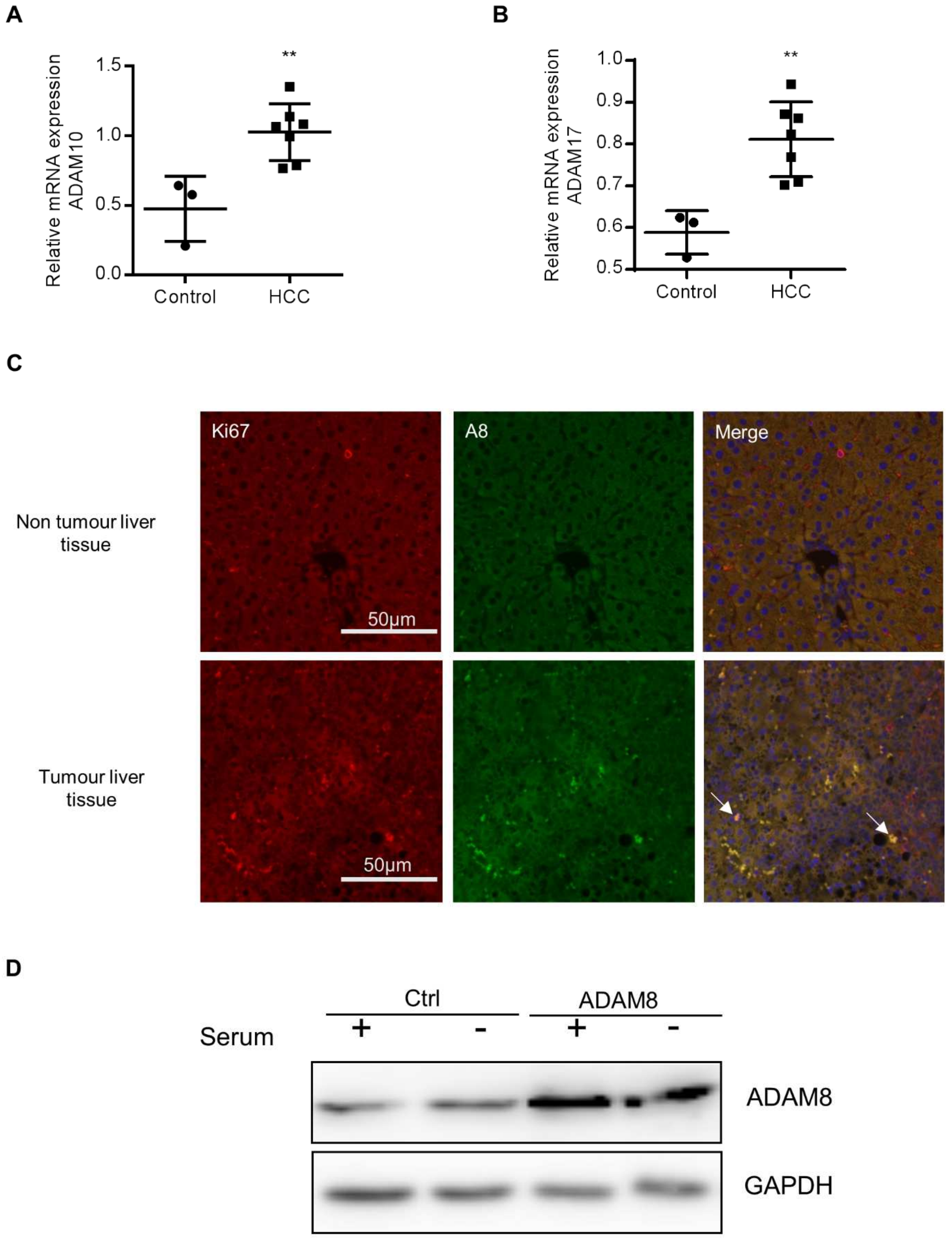

Supplementary Figure 1: mRNA expression of ADAM10 and ADAM17 in murine hepatocellular carcinoma tissues and immunofluorescence analysis of HCC tissue

A-B: The mRNA expression of ADAM10 (A) and ADAM 17 was analysed in murine HCC tissues and compared with control groups. **C:** Cryosections of DEN-induced HCC mice livers were subjected to Ki67 and ADAM8 staining. Total nuclei were counter stained with DAPI. Co-stained areas are indicated with arrows. Scale bars indicate 50µm. **D:** HepG2 cells were treated with control (Ctrl) or ADAM8 over expression vector (ADAM8) and seeded in 6-well plate. Cells were serum starved for 18 h and then analysed for the expression of ADAM8 by Western blot. All quantified data are shown as mean ± SD. * $p < 0.05$, ** $p<0.01$, *** $p<0.001$

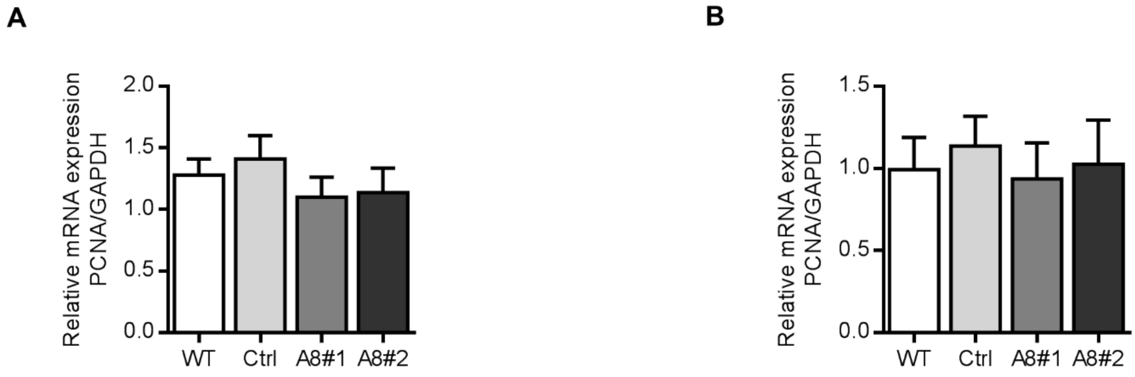

Supplementary Figure 2: mRNA expression of PCNA in hepatoma cell lines

A-B: mRNA expression of PCNA was measured by quantitative PCR. The differences between control and ADAM8 knock down groups were not prominent for both Hepa1-6 (A) and HepG2 (B) cell lines.

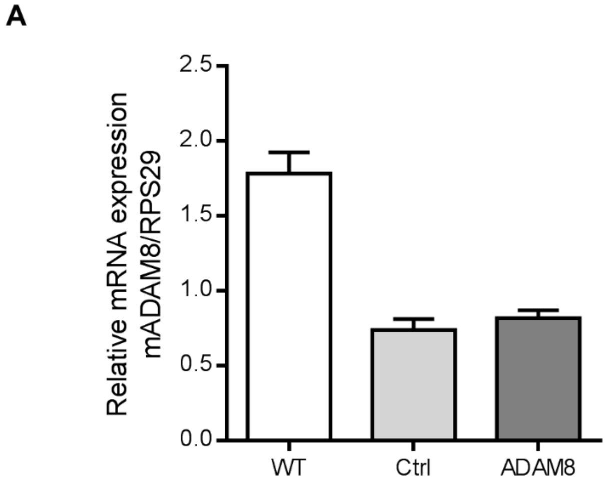

Supplementary Figure 3: mRNA expression analysis of indigenous ADAM8 in Hepa1-6 cells after transfection with hADAM8 overexpression vector

A: mRNA expression of ADAM8 was analysed by qPCR and controlled by RPS29 housekeeping gene. The indigenous expression of ADAM8 in Hepa1-6 cells was same after the transfection in control and ADAM8 over-expression groups of cells.

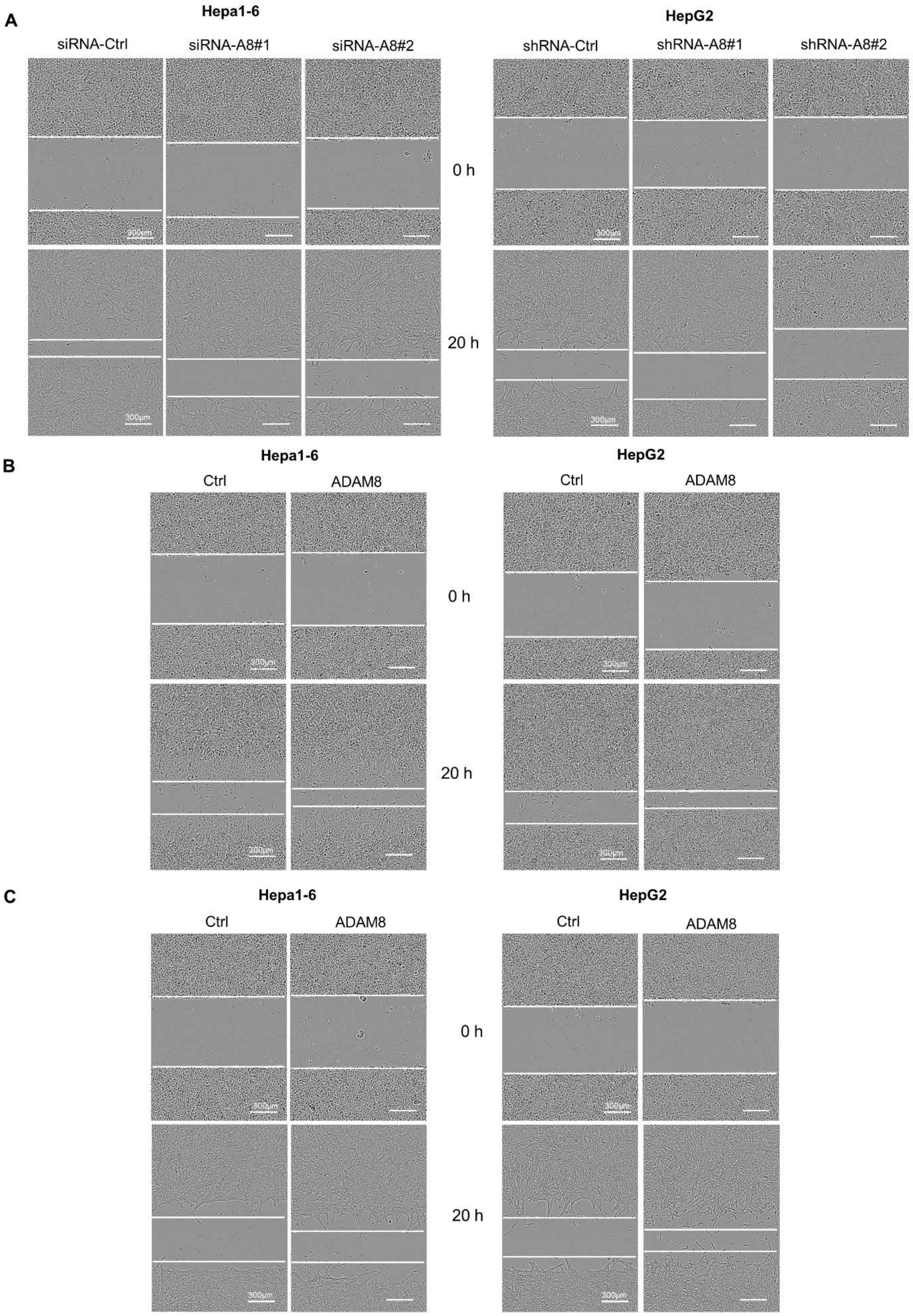
A
Hepa1-6
siRNA-Ctrl
siRNA-A8#1
siRNA-A8#2
HepG2
shRNA-Ctrl
shRNA-A8#1
shRNA-A8#2
0 h
20 h
300μm
B
Hepa1-6
Ctrl
ADAM8
HepG2
Ctrl
ADAM8
0 h
20 h
300μm
C
Hepa1-6
Ctrl
ADAM8
HepG2
Ctrl
ADAM8
0 h
20 h
300μm

Supplementary Figure 4: Representative micrographs for migration and invasion of hepatoma cells

A: Hepa1-6 and HepG2 cells treated with two sequences of siRNA or two shRNA vectors, respectively, for ADAM8 knockdown or non –targeting controls. Representative micrographs for the cell invasion into matrigel at 0 h and 20 h after scratch induction are shown **B-C:** Hepa1-6 and HepG2 cells were transduced with vector for ADAM8 over-expression or control vector. Representative micrographs for cell migration (B) and invasion into matrigel (C) at 0 h and 20 h after scratch induction are shown.

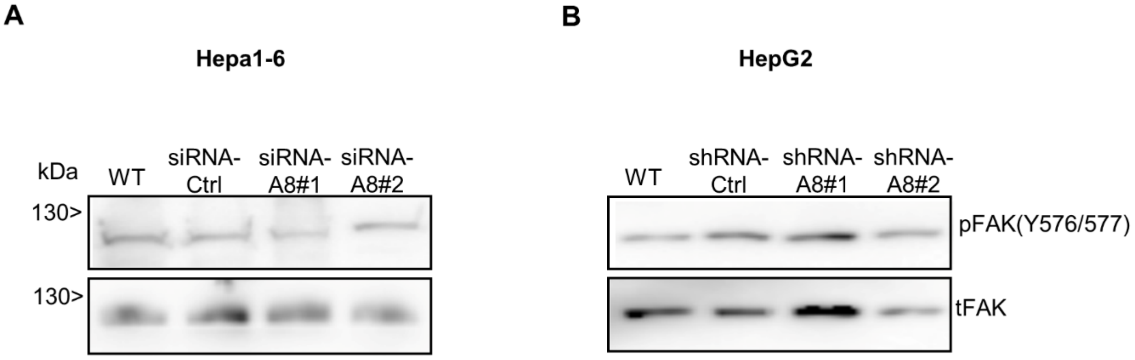

Supplementary Figure 5: Phosphorylation of FAK at Y576/577 remain unchanged after silencing of ADAM8.

A-B: Hepa1-6 and HepG2 cells were analysed for expression of phosphorylated FAK at tyrosine 576/577 and total FAK by Western blotting. Phosphorylation of FAK at this site was unaffected by ADAM8 knockdown in both cell types. Representative blots are shown.

Supplementary table 1: Antibodies, reagents and commercial kits used in the experiments

**Antibodies, reagent and Kits**	**Company**
Rabbit monoclonal ADAM8 antibody (EPR14612)	Abcam (Cambridge, UK)
Rabbit polyclonal ADAM8 antibody	Lifespan Biosciences (Washington, USA)
Rabbit monoclonal p38 antibody (D3F9)	Cell Signalling (Danvers, NA, USA)
Mouse monoclonal β1 integrin antibody (12G10)	Santa Cruz Biotechnology (Dallas, TX, USA)
Rabbit monoclonal pFAK antibody (Y397) (31H5L17)	Santa Cruz Biotechnology (Dallas, TX, USA)
Mouse monoclonal GAPDH integrin antibody (GA1R)	Thermo Scientific (Waltham, MA USA)
Rabbit polyclonal p38 antibody	Cell Signalling (Danvers, NA, USA)
Rabbit polyclonal c-Src antibody	Cell Signalling (Danvers, NA, USA)
Rabbit polyclonal p-Src antibody (Y416)	Cell Signalling (Danvers, NA, USA)
Goat polyclonal pFAK antibody (Y577/576	Santa Cruz Biotechnology (Dallas, TX, USA)
Rabbit polyclonal pFAK antibody (Y925)	Santa Cruz Biotechnology (Dallas, TX, USA)
Rabbit polyclonal FAK antibody	Santa Cruz Biotechnology (Dallas, TX, USA)
Peroxide conjugated secondary antibodies	Jackson Immuno Research (Hamburg, Germany)
LPS (E. coli 0127:B8)	Sigma-Aldrich (Munich, Germany)
RNA extraction RNeasy kit	Qiagen (Hilden, Germany)
PrimeScript RT Reagent Kit	Takara Bio Europe (St-Germain-en-laye, France)
Syber Premix Ex Taq II	Takara (Fitchburg, WI, USA)
Mouse monoclonal Ki-67 antibody (SP6)	Abcam (Cambridge, UK)

Supplementary table 2: qPCR with indicated primer sequences for specified genes was run at 40 cycles of 10 s denaturation at 95 °C, followed by 30 s annealing at indicated temperatures and 15 s amplification at 72 °C.

**Gene**	**Reverse s 5'- 3'**	**Forward 5'- 3'**	**Annealing**
hADAM8	aagcagccgtgcgtcatc	aacctgtcctgactattccaaatctc	62 °C
hGAPDH	ccagccccagcgtcaaaggtg	agggccgatcatggagtctt	60 °C
hPCNA	gaagcaccaaaccaggag	cacaggaaattacaacagca	59 °C
mAdam8	gcgagtgctggaggttgtaa	acccccgtgataagttgcac	64 °C
mAdam10	agcaacatctggggacaaac	tggccagattcaacaaaaca	57 °C
mAdam17	aaaccagaacagacccaacg	aacgaatcgaaccctgactggca	57 °C
mPcna	cccagaacaggagtacag	ggctcattcatctctatgg	59 °C
mGapdh	ggcaattcaacggcacagt	agatggtgatgggcttccc	63 °C
mRps29	cctttctcctcgttgggc	gagcagacgcggcaa	61 °C
